# Recent Cases of Nerve Stretching

**Published:** 1882-02

**Authors:** 


### RECENT CASES OF NERVE STRETCHING.

Stretching of the brachial and sciatic nerves for tetanus and locomotor ataxia has become so common that little attention is now paid to reports of such operations. Very recently the buccal nerve has been stretched for neuralgia, by Dr. A. Wolfler, first assistant in Billroth's clinic. Benedikt has reported a case of stretching of the facial for long standing paralysis with secondary *tic spasmodique*. The *tic* was relieved and the paralysis improved, although the nerve was much degenerated and embedded in cicatricial tissue. M. Le Dentu has stretched the lingual nerve for neuralgia located in the temporal region, auricle, lower jaw, and left side of the tongue. The pain had lasted for five years and had become insupportable. With the view of relieving the pain in the tongue, M. Le Dentu stretched the lingual nerve, and two weeks after the operation the pain had nearly entirely disappeared. M. Polaillon has stretched the inferior dental nerve for violent neuralgia, with complete success. MM. Marcus and Wiet (*Le Prog. Med.*, No. 21,) have experimentally stretched the pneumogastric in rabbits with the following results:—The first animal died three days after the operation, presenting on section all the signs of asphyxia. On the second rabbit, both pneumogastrics were stretched centrically. There was first, congestion, followed in a few minutes by contraction of the vessels of the ear, giving place again shortly to intense congestion. There was also dilatation of both pupils. On the second day after the operation sugar was detected in the urine, and a similar result followed the operation in another rabbit. The second rabbit operated upon had dyspnœa on the day after the operation which progressively increased. The authors believed the animal would ultimately die of asphyxia.

Panas has stretched the frontal nerve for painful blepharospasm with entire relief.

